# The Hospital City

**Published:** 1905-12-02

**Authors:** 


### HOSPITAL ADMINISTRATION.

### CONSTRUCTION AND ECONOMICS.

### THE HOSPITAL CITY.

A GLANCE INTO THE FUTURE.

By Sir Henry Burdett, K.C.B.

For nearly forty years I have been actively working at the administration, finances, construction, general control and inspection of hospitals and kindred institutions throughout the world. I realise, as the medical profession are realising, that the atmosphere of a great city grows less and less suitable to the rapid and complete recovery of patients, who may undergo the major operations, or be suffering from severe and acute forms of disease. Asepsis it is true has reduced the average residence in hospitals from thirty-five days to less than twenty days. It has thereby added probably quite 1,000,000 working days each year to the earning power of the artisan classes in London alone. Medical opinion is more and more favouring the provision of convalescent or suburban hospitals to which patients suffering from open wounds may be removed from the city hospitals. This course is advocated to overcome the difficulty arising from the fact, that in many cases, patients cease to continue to make rapid progress towards recovery, after the seventh or ninth day's residence in a city hospital. A change in such cases to the country restores the balance and completes the recovery, with a rapidity often remarkable.

Thinking out the problem here presented in all its bearings, realising the great and ever increasing cost of sites for hospitals in great cities, the heavy consequential taxes and charges which they have to meet there, and all the attendant disadvantages and drawbacks, I have looked ahead into futurity, and, following the example of the modern pressmen, I venture upon an anticipation in regard to the future of our hospitals, which I hope is intelligent and well founded. Nearly every difficulty in regard to the cost of hospitals and in respect to all the many problems presented by securing the material required, under present systems, for the efficient training of students and nurses, would be removed by this system, which, I foresee, must ultimately be pursued by every intelligent community in the civilised world. Why should we not have, on a carefully selected site well away from the contaminations of the town, and adequately provided with every requisite demanded from the site of the most perfect modern hospital, which the mind of man can conceive, "The Hospital City"? Here would be concentrated all the means for relieving and treating every form of disease to the abiding comfort of all responsible for their adequacy and success. At the present time all the traffic and all the citizens give way to fire engines and the ambulance in the public streets. Necessarily the means of transit to and from "The Hospital City" and its rapidity, would be the most perfect in the world. So the members of the medical staff, the friends of the patients, and all who had business in "The Hospital City," would find it easier and less exacting in time and energy, to be attached to one of the hospitals located therein, than to one situated in the centre of a big population in a crowded town. To meet the urgent and accident cases a few Receiving Houses, or outpost relief stations would be situated in various quarters of the working city, where patients could be temporarily treated, and whence they could be removed to "The Hospital City" by an efficient motor ambulance service.

I can see "The Hospital City" established, can realise the comfort it will prove in practice to the medical profession, to the patient's friends, to those who have to manage the hospitals and train the medical and nursing students, and indeed to all who may go there as well as to the whole community. The initial cost of hospital buildings would be reduced at once to a quarter or less of the present outlay. The cost of administration and working must be everywhere reduced to a minimum. The hygienic completeness of the whole city, its buildings and appliances, must expedite recovery to the maximum extent. In all probability the removal of the sick from contact with the healthy would tend, in practice, to so increase the healthiness of the town population, *i.e.* of the workers in the city proper, as to free them from some of the most burdensome trials which now cripple their resources, and diminish materially the happiness of their lives. Probably the United States, (where a city has sometimes sprung up in twelve months), may be the home where this ideal of mine may first find its realisation in accomplished fact. I may never live to see such a city in actual working or in its entirety, but I make bold to believe its adoption, will one day solve, the more difficult of the problems involved in the curing of the sick in crowded communities.

I have formulated the idea because it seems desirable to encourage an all round discussion as to the best method of checking the growing tendency of the present day to make hospital buildings everywhere too costly. If the idea of "The Hospital City" commends itself to the profession and the public, the practice of treating all the hospital accommodation in each city as a whole will gradually increase and spread, until most of the present pressing difficulties may disappear altogether. That is a consummation devoutly to be wished.

